# Mental Health Issues Among School Children and Adolescents in India: A Systematic Review

**DOI:** 10.7759/cureus.61035

**Published:** 2024-05-25

**Authors:** G. Balamurugan, Sanjay Sevak, Kusum Gurung, M. Vijayarani

**Affiliations:** 1 Psychiatric Nursing, National Institute of Mental Health and Neurosciences (NIMHANS), Bengaluru, IND; 2 Mental Health Nursing, All India Institute of Medical Sciences, Jodhpur, Jodhpur, IND; 3 Mental Health Nursing, Ramaiah Institute of Nursing Education and Research, Bengaluru, IND; 4 Psychiatric Nursing, Employees' State Insurance Corporation (ESIC) College of Nursing, Bengaluru, IND

**Keywords:** india, mental health, adolescent, child, school

## Abstract

Childhood and adolescence are critical developmental stages for mental health, and the environment in which they grow has an impact on their well-being and growth. This study aims to assess mental health issues among school children and adolescents in India. A systematic search was conducted on the literature published between January 2013 and August 2023 in PubMed, Scopus, Cochrane Library, and Eric database. Thirty-one studies with a sample size of 30,970 were included in the final quantitative synthesis, of which 14,381 were male. The overall mean age of the school children and adolescents was 14.58 years, with a standard deviation of 1.35. A diverse range of mental health concerns have been documented in school children and adolescents, exhibiting differing degrees of severity and frequency. The analysis showed that depression was the most prevalent mental health issue among children, followed by social, behavioral, and emotional problems, anxiety, psychological distress, internet technology addiction, stress, social phobia, sexual and emotional abuse, violence, and attention deficit hyperactive disorder. The study concludes that school mental health research in India is critical for personalizing interventions to the specific requirements of the diverse student population, decreasing stigma, and enhancing overall student well-being within the cultural and educational context of the country.

## Introduction and background

India is the world's most populous nation, surpassing China, with a population of 1,425,775,850 [1]. According to UN estimates and global population projections, India's population is predicted to continue expanding for several decades [2]. India has the most children and adolescents worldwide, with 34.8% of the population aged 0 to 19 in 2021 [3,4].

Childhood and adolescence are key developmental phases for mental health, and the quality of their environment influences their well-being and growth [5]. A variety of disorders resulting from deficiencies in a child's physical, cognitive, or behavioral functioning are referred to as developmental disabilities [6]. Attention deficit hyperactivity disorder (ADHD), sensory impairments (loss of hearing and vision), cerebral palsy, epilepsy or seizures, intellectual disability or other learning disorders, autism spectrum disorder (ASD), depression, anxiety, and behavioral problems are common in affected children and adolescents [7,8].

As per a survey conducted in early 2021 by UNICEF and Gallup, children in India were hesitant to seek help for mental stress, with only 41% of young people aged 15 to 24 in India saying it is helpful to seek help for mental health concerns [9]. Globally, 10% of children and adolescents have a mental illness, and one in every seven children 10- to 19-year-olds has a mental disorder, making up 13% of the worldwide disease burden in this age group [5,8]. Furthermore, in 2016, 52.9 million children under the age of five (54% of them were boys) had developmental problems, and around one in every hundred children had autism. Ninety-five percent of these children were from low- and middle-income countries [10,11]. As stated in the publication of the State of the World's Children Report 2021, the South Asia areas, East Asia, and the Pacific had the largest number of teenagers with mental problems [12].

According to Malhotra et al., the prevalence rate of mental health issues in children and teenagers in India was determined to be 23.33% in school and 6.46% in the community [13]. Aside from these studies, the National Mental Health Survey 2016 found that teenagers had a 7.3% prevalence of illness, distributed equally across boys and girls. However, it was greater in metropolitan metro areas, and the prevalence of anxiety issues was 3.6%, with depression-related conditions at 0.8% [14].

Children and adolescents need to be nurtured, and it is the responsibility of all parties involved, parents, educators, the government, legislators, and society at large to support their mental health [15]. Failure to address adolescent mental health disorders has long-term implications on one's physical and emotional well-being, as well as limits one's ability to lead a satisfying adult life [8]. There is enough evidence to support the inclusion of mental health education where the integration can promote children's and teenagers' positive mental health if it is founded on evidence-based practice [15].

School children face many issues, including the inability to seek help when they need it, the inability to trust others, particularly if they have had negative experiences at home, and their unwillingness to disclose problems in school for fear of being victimized. Other obstacles could include a lack of a designated physical setting, non-cooperation from school administration, infrequent availability of a counselor on campus, and a lack of privacy [15]. School children experiencing anxiety and depression can have a significant impact on school attendance and academic performance [8]. Whenever children are identified as having potential mental health issues, they are frequently referred to mental health professionals for treatment. Though well-intentioned, this strategy is ineffective mainly if families encounter obstacles, including language barriers, high cost, insufficient transportation, or job inflexibility that prevents them from keeping consultations [15]. Schools and other learning environments can meet children's and teenagers' mental health and psychosocial well-being requirements, especially in emergencies [12].

Given the growing concerns and prevalence of mental health problems among school children in India, the current review is imperative. The necessity for a thorough examination of the mental health problems in school children in India cannot be overstated. The intense academic pressure, cut-throat competition, and societal pressures faced by school children in India make it crucial to comprehend the extent of their struggles and their impact on their overall well-being. Conducting a systematic review will enable us to gather and examine existing research to gain a holistic understanding of the mental health challenges confronted by school children in India. Through this study, we aim to gain invaluable comprehension of the unique mental health obstacles encountered by school students in India. By doing so, we can pave the way for tailored interventions and support structures to be established. This research is crucial in equipping policymakers, educators, and mental health experts with the knowledge to prioritize and tackle mental health concerns in schools.

Furthermore, the results of this comprehensive analysis have the potential to raise awareness among parents and caregivers, empowering them to identify mental health red flags in their children and seek prompt assistance when necessary. This study was initiated due to the pressing concern of mental health among school-going children in India. These young individuals are faced with a multitude of challenges and pressures that have the potential to impact their well-being significantly. We must gain a thorough understanding of these issues through a systematic review to address and mitigate their effects. By comprehending the scope and nature of these challenges, we can develop targeted interventions and support systems backed by evidence and tailored to the unique needs of these school children, ultimately promoting their holistic development.

## Review

Material and methods

The research includes a systematic review, a methodology established by the Evidence for Policy and Practice Information Centre (EPPI) Centre at the University College London Institute of Education. Preferred Reporting Items for Systematic Reviews and Meta-Analyses (PRISMA) recommendations were also considered to provide transparency, validity, replication, and updateability in our work [16].

Search Strategy

For this review, a combination of search terms (((school[Title]) AND (india[Title])) AND ((child*[Title]) OR (adoles*[Title]))) AND ((((health) OR (issue*)) OR (disorder*)) AND (mental)) was searched on Pubmed. On the Cochrane databases, the following keywords were used: (school[Title]) AND ((child*[Title]) OR (adoles*[Title])) AND (mental) AND ((health) OR (issue*) OR (disorder*)) AND (india[Title]). We reviewed the Scopus database using combinations of the search terms (school) AND (child) OR (adolescent) AND (mental) AND (health) OR (issue) OR (disorder) AND (India). For the Education Resources Information Center (ERIC) database, the search terms included school mental health issues in India, and the filter applied was studies conducted in India, journal articles, and the years range from 2014 to 2023.

Inclusion and Exclusion Criteria

Studies included in the analysis focused on participants aged five to 19 years, with literature published between January 2013 and August 2023. Only full-text studies available in English were considered, encompassing both online and offline research on mental health issues in school children. The studies needed to recruit participants solely from India could be observational studies (such as case-control studies, surveys, and cohort studies) or intervention studies with baseline prevalence data presented.

Exclusion criteria ruled out studies focusing on populations below five or above 19 years, those including school children with other psychiatric disorders and substance abuse issues, articles lacking methodological details, reviews (both literature and rapid reviews), case reports, commentaries, editorial letters, books, conference abstracts, and guidelines.

Selection process

**Figure 1:**
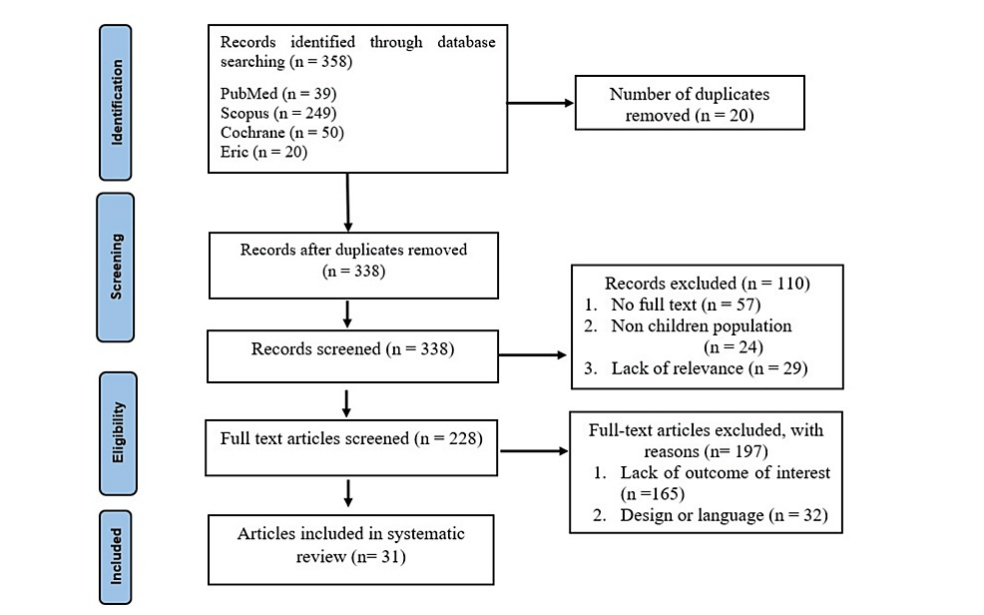
PRISMA flow diagram for the selection process of studies systematic review PRISMA: Preferred Reporting Items for Systematic Reviews and Meta-Analyses

The initial phase of the search revealed 358 articles from indexed journals: 39 from the PubMed database, 249 from Scopus, 20 from ERIC, and 50 from Cochrane. For the initial screening, data like author, title, year, study design, age category, variable measured, methodology, and abstracts were gathered and entered into a spreadsheet. The two reviewers (SS, KG) independently extracted data from all the pertinent studies to eliminate redundant documents. Precisely 20 were duplicates and were thus eliminated, yielding 338 in total. The abstracts of the 338 publications were subjected to the inclusion and exclusion criteria. Consequently, 228 articles satisfied the inclusion requirements and were initially qualified for evaluation. Then, the research articles were downloaded to be thoroughly examined. The reviewers separately analyzed the papers and extracted the most pertinent material, which was then entered into a spreadsheet. Then, data were re-checked, and discrepancies were resolved through concord (GB, VM) against inclusion and exclusion criteria. This initial evaluation and discussion of the 228 publications' studies excluded 197 papers that did not fit the inclusion criteria. As a result, 31 papers were ultimately chosen for analysis, as illustrated in Figure 1.

Data Analysis

To analyze the studies, the researchers organized the most key data for the review's objective using an analytical grid. First, data was classified using an inductive technique. Second, researchers compared their findings to establish an agreement on the key conclusions of the review.

Results

The study aimed to identify original research about mental health issues among school children and adolescents aged 19 years and under in India from studies published predominantly between January 2013 and August 2023. To obtain relevant articles, our initial literature search yielded 358 articles, 31 of which were included in this systematic review, the characteristics of which are summarized in Table 1.

**Table 1:** Summary of the studies evaluating the profile of mental health issues in school children SSRQ: Short Self-Rating Questionnaire, SDQ: Strengths and Difficulties Questionnaire, SPIN: Social Phobia Inventory Tool, KADS: Kutcher Adolescent Depression Scale, GHQ 12: General Health Questionnaire, PHQ-9: Patient Health Questionnaire, IAT-A: Internet Addiction Test-Adolescents, GAS-SV: Game Addiction Scale-Short Version, SAS-SV: Smart/mobile phone Addiction Scale-student version, TAS: Television addiction Scale, RCADS: Revised Child Anxiety and Depression Scale - Short Version, TCI: Temperament and Character Inventory, FAD: McMaster Family Assessment Device, PHQ-9A: Patient Health Questionnaire for Adolescents, GAD-7: Generalised Anxiety Disorder, MFQ: Short mood and feelings questionnaire, SCARED: Screen for Child Anxiety and Emotion-related Disorder, SMFQ: Short Mood & Feeling Questionnaire, GSHS: Global School Based Student Health Survey questionnaire, BDI: Beck Depression Inventory, CDI: Children's Depression Inventory, CES-DC: Center for Epidemiological Studies-Depression Scale for Children, CATS: Children's Automatic Thoughts Scale, CTI-C: Cognitive Triad Inventory for Children, SAAS: Scale for Assessing Academic Stress, A-COPE: Adolescent Coping Orientation to Problems Experienced Inventory, SPSI-R: Social Problem Solving Inventory-Revised-Short Form, SDSQ - Sociodemographic Sheet and Questionnaire, ISPCAN: International Society for the Prevention of Child Abuse and Neglect, ICAST-CI: Child Abuse Screening Tool - Children’s Institutional Version, TSQ: Teenage Screening Questionnaire-Trivandrum

Author (year)	Place	Study design	Age, years (mean±SD or range)	Sample size (boys, %)	Variables measured	Tools used	Methodology
Ray et al., 2022 [17]	Delhi and Mathura (UP)	Descriptive cross-sectional study	9 to 19 years	369 (218,59.07%)	Stress	SSRQ	Random sampling method
Nath et al., 2022 [18]	Assam, India	Descriptive cross-sectional online study	4-17 years (10.39 ± 3.53)	242 (117,48.43%)	Behavioral and emotional difficulties of children	SDQ	Purposive nonprobability sampling
Gupta et al., 2022 [19]	Dehradun, Uttarakhand, India.	Cross-sectional study	12-19 years	600 (324,54%)	social phobia and symptoms of depression	SPIN, KADS	Multistage random sampling technique
Bharati et al., 2022 [20]	Patna City, Bihar	Cross-sectional study	11-19 years	838 (240,29.64%)	Depression	Modified PHQ-9	Systematic random sampling
Rathi et al., 2022 [21]	Kolkata, West Bengal	Cross-sectional, two-randomized-group designed, comparative study	14-17 years	1000 (500, 50%)	Internet addiction, substance use, personality, Conduct and attention disorders, anxiety, depression, autistic-like detachment, and current mental health	IAT, GHQ 12 and CRAFFT 2.0 questionnaire, TCI, FAD, Devereux Scale for Mental Disorders	Purposive sampling
Gonsalves et al., 2022 [22]	Goa, India	Randomized controlled trial	13-19 years (15·61±1·68)	11 (4,36.36%)	Psychosocial problem severity and self-reported depression and anxiety	Youth Top Problems, RCADS-25	Random sampling
Muthusamy et al., 2022 [23]	Tiruchirappalli, South India	Cross-sectional questionnaire survey	13-17 years	550 students (266,48%)	Anxiety	SCARED child Version	Stratified random sampling
Jeelani et al., 2022 [24]	Kashmir, India	Cross-sectional study	15-19 years (17.5 ±1.26)	426 adolescents (242,56.8%)	Depression and anxiety	PHQ-9A, GAD-7	Purposive sampling method
Mathew et al., 2022 [25]	Chennai, Tamil Nadu	Survey	5-16 years	874 (435,49.8%)	Depression	MFQ	Voluntary basis
Amudhan et al., 2021 [26]	Kolar district, Karnataka	Cross-sectional survey	(12.58±0.97)	1729 (732,42.33%)	Internet addiction, game addiction, smartphone addiction, and television addiction	IAT-A, GAS-SV, SAS-SV, TAS	Stratified cluster sampling
Das et al., 2021 [27]	Udupi taluk, Karnataka	Cross-sectional study design	14-16 years, (14.43± 0.59)	659 (386,58.6%]	Adolescents' behavior and mental problems	SDQ	Two-stage stratified clustered sampling
Kothari et al., 2021 [28]	Tehri district of Uttarakhand	Cross-sectional study	11-17 years, (14.66±1.88)	150 (76,50.7%)	Social and behavioral problems	SQD	Non-probability convenient sampling
Kirubasankar et al., 2021 [29]	Puducherry, India	Cross-sectional, comparative study	14-18 years, (15.85+/- 0.7)	462 (225,48.7%)	Anxiety disorders	SCARED scale	Stratified cluster random sampling.
Bairwa et al., 2021 [30]	Jaipur, Rajasthan	Cross-sectional study	6-16 years	301 (161,53.49%)	Depression	SMFQ	Snowball sampling technique
Dangi et al., 2021 [31]	Rohtak, Haryana	cross-sectional study	14-17 year	400 (213, 53.2%)	Emotional and behavioral problems	SDQ	Convenience sampling
Mangal et al., 2020 [32]	Jamnagar, Gujarat	Cross-sectional study	10-19 years	742 (0,0%)	Common mental health problems.	GHQ‑12	Multistage stratified random sampling technique
Beattie et al., 2019 [33]	Vijayapura and Bagalkote districts, Karnataka	Cross-sectional survey	13-14 years (Median age 13)	1191 (0,0%)	Feeling about hope, depression, sexual abuse, eve teasing, and emotional support	Socioeconomic factors, gender disadvantage factors	Cluster randomized
Shukla et al., 2019 [34]	Barabanki, Uttar Pradesh	Cross‑sectional study	10-19 years	2187 (0,0%)	Depression	KADS	Multistage sampling
Puwar, et al., 2018 [35]	Sabarkantha districts, Gujarat	Cross‑sectional study	11-19 years (14.2±1.4)	477 (294,61.63%)	Mental health	SDQ	Not specify (school randomly selected)
George et al., 2018 [36]	Ramanagara district, Karnataka.	Cross-sectional study	10-19 years (13.12±1.47)	449 (157,35%)	Psychosocial problems	SDQ	Not specify
Harikrishnan et al., 2017 [37]	Tezpur, Assam	cross-sectional design	13-17 years (14.81± 1.117)	1403 (734,52.3%)	Emotional and behavioral problems	SDQ	Convenience sampling
Jha et al., 2017 [38]	Patna, Bihar	Cross‑sectional study	14-18 years (16.04 ± 1.24 years)	1412 (893, 63.2%)	Depression	BDI-II	Two-stage cluster sampling method
Kumar et al., 2019 [39]	Thrissur, Kerela	Cross-sectional study.	13 to 16 and above	6682 (4242, 63.5%)	Sexual, physical, and emotional abuse	ISPCAN, ICAST-CI	Random sampling
Nair et al., (2017) [40]	Anand, Gujarat	cross-sectional study	13-17 years (median age 15 years)	693 (356, 51.31%)	Emotional problems, conduct problems, hyperactivity, peer problems, prosocial behavior	SDQ, TSQ	Random sampling
Ramya et al., 2017 [41]	Bengaluru, Karnataka	Cross-sectional study	School children of 5-12 years	3120 (1770,56.7%)	Attention deficit hyperactivity disorder	Conner’s teacher rating scale and parents rating scale	Convenience sampling method.
Deb et al., 2017 [42]	Puducherry	Cross-sectional study	14-17 years	291 (121, 41.6%)	Global mental health, psychological distress, anxiety, and loss of behavioral/emotional control	Mental Health Inventory	Not specified
Deb et al., 2016 [43]	Kolkata, West Bengal	Cross-sectional study	15-18 yrs, (16.65± 0.67)	370 adolescents (182, 49.2%)	Mental health	Self-concept scale, Beck anxiety inventory, social adjustment inventory	Not specified
Singhal et al., 2016 [44]	Bangalore, Karnataka	Cross-sectional study	13-18 (14.4 years± 0.67)	800 (344,43%)	Depression severity, problems, and felt needs of adolescents	SDSQ, CDI, CES‑DC, CATS, SPSI‑R, and A‑COPE.	Voluntary participation
Singhal et al., 2014 [45]	Bangalore, Karnataka	Intervention study	13-18 years (14.4-14.5)	300 (54,18%)	Coping Skills, depressive symptoms, negative cognitions, academic stress, and social problem-solving	CDI, CES-DC, CATS, CTI-C, SAAS, A-COPE, SPSI-R, and a feedback questionnaire.	Random sampling
Roy et al., 2014 [46]	East Delhi	Cross-sectional study	13-16 years	400 (200, 50%)	Psychological distress	GHQ-12	Stratified randomization sampling technique
Rani et al., 2013 [47]	Chennai City, Tamil Nadu	Cross-sectional study	12-18 years	1842 (895, 47.20%)	Mental Health symptoms and substance use	GSHS questionnaire	Simple random sampling

The systematic review includes 24 cross-sectional studies [19,20,23,24,26-28,30-44,46,47], two descriptive cross-section studies [17,18], two comparative cross-section studies [21,29], whereas one randomized control trial [22], one survey study [25], and one intervention study [45] respectively. This review had 30,970 participants, of which 14381 (46.43%) were male. The overall mean age of the school children and adolescents was 14.58 years, with a standard deviation of 1.35 [22,24,26-29,35-38,43,44]. As per participants' concern, the sample size for cross-section studies ranging from 150 to 6682 participants, 242-369 participants for descriptive cross-section studies [17,18] and 462-1000 participants for comparative cross-section studies [21,29], whereas in randomized control trials [22], survey study [25], and intervention study [45], number of participants were 11, 874, and 300 respectively.

Out of a total of 31 studies, the maximum number of studies, i.e., seven studies from Karnataka [26,27,33,36,41,44,45], three studies from Tamil Nadu [23,25,47], and Gujrat [33,35,40], two studies each from Delhi [17,46], Bihar [20,38], Assam [18,37], Uttarakhand [19,28], Pondicherry [29,42], and West Bengal [21,43], and rest of the studies one each from Uttar Pradesh [34], Haryana [31], Kerala [39], Rajasthan [30], Kashmir [24], and Goa [22]. These results highlight the range of research efforts in the states and territories mentioned, emphasizing their geographical diversity.

The Strengths and Difficulties Questionnaire (SDQ) was a validated tool used to examine mental health difficulties [18,27,28,31,35-37,40] used by eight studies, General Health Questionnaire (GHQ) [21,32,46], and Kutcher, Adolescent Depression Scale (KADS) [19,34], used by three studies respective, whereas, Child Anxiety and Emotion-related Disorder (SCARED) [23,29], and Mood & Feeling Questionnaire (MFQ) [25,30], used by two studies, Short Self-Rating Questionnaire (SSRQ) [17], Social Phobia Inventory Tool (SPIT) [19], Patient Health Questionnaire (PHQ) [20], Internet Addiction Test-Adolescents (IAT) [21], Revised Child Anxiety and Depression Scale - Short Version (RCAD) [22], Patient Health Questionnaire for Adolescents (PHQA) [24], Generalised Anxiety Disorder (GAD) [25], Beck Depression Inventory (BDI) [38], Trivandrum Screening Questionnaire (TSQ) [40] were used by one study respectively.

The studies examined various aspects of mental health by implementing various validated tools, demonstrating a comprehensive approach to assessment. This diversity in assessment methods strengthens our understanding and evaluation of mental health difficulties.

Quality Assessment

A checklist derived from the Joanna Briggs Institute (JBI) was utilized to evaluate the quality and risk of cross-sectional research and other studies in preparation for systematic reviews [48]. While most studies suggested a low risk of bias, most scored five or higher. The evaluation findings are shown in Table 2.

**Table 2:** Results of systematic review using the JBI critical appraisal tools for cross-section studies, quasi-experimental studies and randomized control trials U: unclear, NA: not applicable, CSS: cross-section studies, QES: quasi-experimental studies, RCT: randomized controlled trials; JBI: Joanna Briggs Institute

Authors (years)	Study type	Clearly defined criteria for inclusion of sample	The subjects of study and setting explained in detail	The valid and reliable way to measure exposer	Criteria and objective, standard used for condition measurement	Identified confounding factors	Confounding factors dealing with strategies stated	The valid and reliable way to outcomes measured	Used appropriate statistical analysis	JBI Score	Bias Risk
Ray et al., 2022 [17]	CSS	+	+	+	+	U	–	+	+	6	Low
Nath et al.,2022 [18]	CSS	+	+	+	+	+	–	+	+	7	Low
Gupta et al., 2022 [19]	CSS	+	+	+	+	+	–	+	+	7	Low
Bharati et al., 2022 [20]	CSS	+	+	+	+	+	–	+	+	7	Low
Rathi et al., 2022 [21]	CSS	+	+	U	+	–	–	+	+	5	Minor
Gonsalves et al., 2022 [22]	RCT	Assessed with the JBI appraisal checklist for randomized control trials, found low risk for bias									
Muthusamy et al., 2022 [23]	CSS	+	+	+	+	–	–	+	+	6	Low
Jeelani et al., 2022 [24]	CSS	+	+	+	+	+	–	+	+	7	Low
Mathew, et al., 2022 [25]	CSS	+	–	+	+	+	–	+	+	6	Low
Amudhan et al., 2021 [26]	CSS	+	+	+	+	–	–	+	+	6	Low
Das et al.,2021 [27]	CSS	+	+	+	+	–	–	+	+	6	Low
Kothari et al. 2021 [28]	CSS	+	+	+	+	+	–	+	+	7	Low
Kirubasankar et al., 2021 [29]	CSS	+	+	+	+	–	–	+	+	6	Low
Bairwa et al., 2021 [30]	CSS	+	U	+	+	+	–	+	+	6	Low
Dangi et al., 2021 [31]	CSS	+	+	+	+	+	–	+	+	7	Low
Mangal et al., 2020 [32]	CSS	+	+	+	+	­–	–	+	+	6	Low
Beattie et al., 2019 [33]	CSS	+	+	+	+	+	–	+	+	7	Low
Shukla et al., 2019 [34]	CSS	+	+	+	+	+	–	+	+	7	Low
Puwar et al., 2018 [35]	CSS	+	+	+	+	+	–	+	+	7	Low
George et al., 2018 [36]	CSS	+	+	+	+	+	–	+	+	7	Low
Harikrishnan et al., 2017 [37]	CSS	+	+	+	+	–	–	+	+	6	Low
Jha et al., 2017 [38]	CSS	+	+	+	+	–	–	+	+	6	Low
Kumar et al., 2019 [39]	CSS	+	+	+	+	–	–	+	+	6	Low
Nair et al., 2017 [40]	CSS	+	–	+	+	–	–	+	+	5	Minor
Ramya et al., 2017 [41]	CSS	+	–	+	+	–	–	+	+	5	Minor
Deb et al., 2017 [42]	CSS	+	+	+	+	–	–	+	+	6	Low
Deb et al., 2016 [43]	CSS	+	+	+	+	–	–	+	+	6	Low
Singhal et al., 2016 [44]	CSS	+	–	+	+	–	–	+	+	5	Minor
Singhal et al., 2014 [45]	QES	Assessed with JBI appraisal checklist for Quasi-experimental study, found low risk for bias									
Roy et al.,2014 [46]	QES	+	–	+	+	–	–	+	+	5	Minor
Rani et al., 2013 [47]	CSS	+	–	+	+	–	–	–	+	4	High

Discussions

The current review represents literature regarding mental health issues among school children in India. The review showed that there is significant heterogeneity in reporting patterns across studies, with some studies investigating specific mental health problems while others reflected combined mental health symptoms. In the current review, a wide range of mental health issues have been identified among school children and adolescents, varying in severity and prevalence. The current review reported a wide range of mental health issues among school children with different severity and prevalence. Among children with mental health issues, depression [19,20,22,24,25,30,34,38,44,45] was more prevalent in the majority of studies, followed by social, behavioral, and emotional problems [18,27,28,31,35-37,40], anxiety [22-24,29], psychological distress [33,46], internet/technology addiction [21,26], stress [17], social phobia [19], sexual and emotional abuse [39], violence and mental health [43], ADHD [41], and other mental health issues [32,42,47]. Moreover, the findings of the review have been synthesized from different parts of the country and at different age groups (school-going/adolescents). Through an in-depth analysis of mental health concerns in school-age children and adolescents, it becomes clear that a wide range of issues exists, varying in severity and prevalence. Particularly, several studies have shown that depression is a prevalent problem in this population, garnering significant attention. Along with this, social, behavioral, and emotional difficulties, as well as anxiety, are also commonly reported as major challenges in mental well-being among this demographic. The research also sheds light on a diverse array of other mental health struggles, such as psychological distress, addiction to technology and the internet, stress, social phobia, sexual and emotional abuse, links between violence and mental health, and ADHD. The vast scope of mental health concerns impacting children and adolescents serves as a powerful reminder of the intricate nature of these issues. It is critical to acknowledge their intricacy and the potential links between them, highlighting the importance of personalized interventions and supportive resources. By acknowledging the widespread and significant impact of these challenges, educators, parents, and policymakers can join forces to implement successful tactics to enhance the overall wellness of young students and adolescents.

The review recommended the development of a multidisciplinary, comprehensible, and highly specialized approach to child and adolescent mental health services that offers mental health care in a flexible, easily accessible, and less stigmatizing way. The treatments must address mental health education, employment, and rehabilitation, in addition to diagnosing and treating mental health issues. Moreover, strengthening educational systems to safeguard and enhance children's and adolescents' mental health and psychosocial well-being so that teachers and other caregivers could get training on identifying children exhibiting common mental health issues and the mental health screening incorporated with school health programs. The current review also suggested that it is critical to have competent, skilled school counselors who can collaborate with mental health professionals. The mental health interventions can range from close coordination among stakeholders, imparting skills at the school level for early detection and intervention, a favorable policy framework, expanding outreach services, and substance abuse awareness and sensitization campaigns for adolescents. Furthermore, ensure that the community, family, and school collaborate constructively to build a safe and supportive learning environment. Regular integration of mental health services in the educational context can be an effective method for fostering positive mental health in school children and adolescents. However, much study is needed on this topic globally, particularly in low- and middle-income nations. The National Mental Health Policy, India (2014) incorporated key strategic areas, which included community participation in mental health issues, efficient governance and delivery mechanisms for mental health, promotion of mental health at schools and Anganwadi centers, prevention of mental illness and reduction of suicide; universal access to mental health services; and research on mental health and allied disciplines [49].

Studies on Depression

Ten studies provided data on the prevalence of depression as the most common mental health issue among school children. The current review reported a wide range of depression prevalence among school children, with different severity and prevalence. Furthermore, 10 studies reported that the overall prevalence of depression was 30.65% (out of 7749 school children) [19,20,24,25,30,34,38,44,45]. The referral rates from depression available studies from India varied range from 12.22% (38 of 301) [20] to 51.52% (429 of 838) [30]. This finding is partly consistent with the findings of Jayashree et al., who indicated that 40.7% of their school children experienced depression [50].

The review further revealed that depression among children and adolescents was significantly associated with female participants, late adolescence, student studies in senior classes (9th-11th), screen time, academic discontent, and parental discord [20]. In terms of gender prevalence, evidence estimated that female adolescents had a greater prevalence of depression than male adolescents, and the same trends continue to be observed with negative cognitions [24,25,38,44]. Furthermore, female adolescents experienced higher depressive symptoms, and the associated factor of depression was reported due to poor social problem-solving skills, negative cognitions, and more problematic interpersonal interactions [44]. Besides, COVID-19 was a stressful situation with unique challenges for everyone, in which depression was found to be linked to a history of COVID-19 infection [24]. Concerning online studies, depression is a higher risk observed in children who attended more than four hours of online classes and those who used a mobile phone for online education than those who used other devices, such as tablets or computers. While children who spent engaging more with family and relatives had a lower risk of depression [25]. Other associated factors revealed that depression was much higher in individuals who lived in rural areas, were in their early and mid-adolescent years, and studied private schools [34].

A significant proportion of school-aged children were depressed, which highlighted the need for reinforced and strengthened school-based mental health screening programs and collaboration with parents or caretakers, teachers, and mental health professionals to deal with problems more effectively [34]. Furthermore, studies suggest mechanisms must be implemented in a similar future pandemic to safeguard children's psychological well-being by providing early and adequate psychological support [25]. Based on a comprehensive analysis of ten research studies, it was determined that 30.65% of school-aged children and teenagers experience symptoms of depression. Various factors contributed to this prevalence, such as gender, being in the later stage of adolescence, higher academic levels, increased screen time, dissatisfaction with academic performance, family conflicts, and stressors related to the COVID-19 pandemic.

Studies on Behavioral, Emotional, and Social Problems

A total of eight studies provided data on the prevalence of behavioral, emotional, and social problems among school children and adolescents. Many behavioral and emotional problems and social occur as a result of their psychosocial needs, varying in severity among school-aged children and adolescents. The SDQ [18,27,28,31,35,36,37,40] was the standardized scale assessment used in all the studies. This systematic review concluded that as per the SDQ total difficulty score, the overall prevalence of behavioral, emotional, and social problems was 14.85% [18,27,28,31,35,36,37,40]. The referral rates reveal a wide variance in behavioral, emotional, and social problems available in studies from India that varied from 6% [40] to 36.3% [27].

The behavioral, emotional, and social problems for school children suggest that conduct disorder [27,31,37] was the most common, followed by hyperactivity [28,31,37], emotional problems [28], and peer problems [27]. However, peer problems appear to be the most important concern for adolescents. Among participants in the COVID-19 epidemic, the most prevalent was peer problems [18,36], followed by conduct problems (25.6%), emotional problems (23.1%), and hyperactivity (11.1%) [18].

The behavioral, emotional, and social problems data suggest that gender has a significant impact on mental health status [28]. The study identified gender disparities in patterns of behavioral and emotional problems among adolescents. Peer problems were more common in boys than in girls, although emotional symptoms were more common in girls [35]. According to the strengths and difficulty questionnaire total difficulty score of emotional and behavioral disorders, more females (8.5%) than boys (2.8%) were in the abnormal category, and the association was statistically significant (p<0.05) [31]. Furthermore, girls experienced more emotional problems, whereas boys had more of the other mental health issues [27,40]. In comparison to boys study participants, girls had higher abnormal scores in all behavioral subsets, with the highest in conduct problems, followed by hyperactivity (92.3%), peer problems (85.7%), and emotional problems (80.0%) [28]. However, on the contrary, conduct and peer problems were more documented in boys than girls during COVID-19 [31]. Except for peer concerns, all mental health issues were shown to be more prevalent among rural children [40]. Moreover, the associated factors behind this were the illiterate mother, occupation of the parents, nuclear family, family history of alcoholism and financial issues in the family, and daily physical punishment [35]. Furthermore, the type of family was significantly associated with the SDQ total difficulty score [18].

The role of gender plays a crucial role in mental health disparities, as girls tend to struggle with emotional symptoms while boys face difficulties with peer relationships. In rural areas, children are particularly affected by these disparities due to various factors such as maternal education, parental occupation, family structure, alcoholism, financial problems, and physical punishment. Therefore, targeted and culturally sensitive interventions must be implemented to address these challenges, considering each individual's unique needs and circumstances.

The present review also suggests that it is essential to provide school counselors who are knowledgeable, skilled, and can collaborate with mental health specialists and professionals [18]. Furthermore, training for both parents and teachers should be provided to offer prompt intervention [18,36]. Regularly integrating mental health services in the educational setting can be an effective strategy for promoting adolescent resilience [31]. There is an urgent need for dealing with emotional and mental health issues among school-aged adolescents. These issues need to be addressed in the Rashtriya Kishor Swasthya Karyakram framework [35].

Studies on Anxiety

Table 1 summarizes studies assessing anxiety in the study population. The overall prevalence of anxiety among school children was 35.66% [23,24,29]. The range for anxiety prevalence was 20% [24] to 51% [23] among school children and adolescents. However, during the COVID-19 pandemic, the anxiety score was 50.7 [22]. The majority of findings have suggested that anxiety was more common in girls than boys [23,24,29]. The combination of extremely ambitious students with a highly competitive exam style may have contributed to the higher prevalence of anxiousness as they approach board exams [23]. The prevalence of anxiety disorders was significantly associated with students who are staying in a hostel, students with employed mothers, and those living in extended family contexts [23,29]. During the pandemic era, anxiety was associated with a history of COVID-19 infection, a family history of COVID-19 diagnosis, and hospitalization due to COVID-19 in the family [24].

Adolescents in urban schools exhibited an increase in incidences of anxiety disorder, as well as any subtypes of anxiety, than students in rural schools [29]. This was opposed by the study conducted by Muthusamy et al. (2022) [23], which documented that anxiety disorders were associated with significantly more reports in students who are studying in rural schools [23]. The varying prevalence of anxiety disorders in the school highlights the need for school-based specific diagnostic screening instruments. The significant burden showed in the need for early interventions to support teenagers, particularly those with a COVID-19 self/family history [24].

The fluctuating rates of anxiety disorders stress the critical need for targeted diagnostic tools designed for school settings. This research underscores the urgency of providing early interventions, especially for adolescents who have been affected by COVID-19, underscoring the significance of mental health programs within schools. These results strongly emphasize the complex layers of anxiety experienced by children in school, urging the implementation of focused approaches to cater to the unique needs of this group.

Studies on Other Mental Health Issues

Table 1 displays miscellaneous studies on other mental health symptoms among school children and adolescents. The study showed that 48.78% of adolescent girls reported three or more symptoms suggestive of mental health problems, such as anxiety, depression, and psychosocial distress, while 15.7% of students felt lonely [32]. Furthermore, 17% of students could not sleep at night most of the days during the last 30 days. Besides, 32% of students reported sadness. Even more, 9.3% of students reported ever using tobacco products, while 3.9% reported ever using alcohol [47]. Furthermore, regarding differences in the mental health status of adolescents, significant disparities in global mental health, psychological distress, and depression were found among socioeconomic groups [42]. Additionally, there is evidence that adolescents receive a lack of support from their caretakers or peers [47].

The focus was on the parental need to regularly monitor their children and strengthen the school's mental health services [47]. Furthermore, there is also a need for advocacy in the fields of welfare, well-being, and protection against violence for children and adolescents [42]. The review also suggested that teachers could get training on how to identify children exhibiting common mental health symptoms, and the mental health screening should be incorporated into the school health program regularly so students are screened for mental health problems at least once a year [32].

The various mental health symptoms discussed in the research call for a holistic approach that involves parents, schools, and broader advocacy efforts to protect the mental well-being of school-aged children and teenagers. The proposed strategies are multifaceted and aim to foster a supportive environment while also improving early identification and intervention for mental health issues in this vulnerable group.

Studies on Psychological Distress

Various studies have focused on psychological distress among school children/adolescents [33,46]. More than one-third (35.1%) of female participants had little hope for the future, while 6.9% reported feeling low, depressed, or hopeless. Furthermore, 1.6% reported sexual abuse, 8.0% experienced recent eve teasing, and 6.3% claimed no emotional support from their parents. The suicidal ideation was linked to both sexual abuse and a lack of emotional support from parents [33]. Around 22% of the girls had some psychological problems, out of which 15.75% had signs of stress and 6.25% had a serious problem. The findings suggest that the emotional distress of 10th-grade students was 2.45 times that of 9th-grade students due to more academic stress among 10th-class students [46]. Thus, there was an immediate need for interventions that are adolescent-friendly and readily available services for girls who need treatment and support [33]. Furthermore, the study's recommendations are for parents, schools, and professionals to work together to alleviate the suffering caused by stress in many of these adolescents [46].

The research emphasizes the pressing need for focused interventions and supportive resources to tackle the emotional struggles experienced by school-aged children and teens. It is essential for all relevant parties to work together to offer timely aid and promote a more positive and nurturing emotional atmosphere for this at-risk group.

Studies on Internet/Technology Addiction

Two studies provided data on internet addiction/technology among school children. Internet addiction/technology has emerged as a significant public health issue in India. The prevalence of technology addiction was 10.69% among school-going adolescents, in which phone addiction is the most common, followed by game addiction [26]. Furthermore, adolescents who were addicted to the internet showed a high level of novelty seeking but a low level of persistence [21]. Technology addiction was found to be more prevalent in boys than in girls and in rural adolescents than in urban adolescents [26]. Studies have shown that internet addiction is associated with conduct problems and depression [21]. Furthermore, adolescents with internet addiction have also been showing greater trouble with problem-solving, communication, affective responsiveness, affective participation, and behavior control in their families [21]. Technology addiction is associated with psychological distress, higher adolescent age, male gender, poor school contacts, parental relationships, dysfunctional families, and parental video game use [21,26].

Since the family plays a significant role in an Indian context, family-focused strategies for dealing with internet addiction must be incorporated [21]. Immediate attention is needed to promote healthy behaviors toward technology; an integrated socio-ecological approach with a multi-level strategy that targets risk factors at various stages is required [26].

The current study highlights the importance of promoting positive technology habits and advocates for a comprehensive socio-ecological approach. This multifaceted approach, which tackles risk factors at various stages, is crucial in effectively addressing the growing problem of internet addiction among Indian school children.

Studies Related to Stress/Social Phobia/and ADHD

Table 1 shows studies that identified stress [17], social phobias [19], and ADHD [41] among school children and adolescents. The effect of coronavirus is not only physical but also psychological on people of all ages, with children being particularly vulnerable. During the COVID-19 epidemic, the study showed that 30.08% of students had low stress levels, while 62.87% and 7.08% had moderate and severe stress levels, respectively [17]. Additionally, evidence regarding social phobia, 23.7% of adolescents experience mild social phobia, followed by 11.5% (moderate phobia) and 2.3% (severe phobia) [19]. In terms of ADHD, it is a worldwide prevalent problem, and it is also one of the most emerging problems in India. The prevalence of ADHD was 1.3%. Among the ADHD-positive cases, children belonging to the hyperactivity type were 34.1%, inattention was 9.8%, and combined type was 56.1%. The prevalence of ADHD in private schools was 1.25%, and in government schools was 1.37% [41]. Females reported higher levels of moderate and severe social anxiety than males [19], while male to female ratio for ADHD was 1.6:1 [41].

The studies suggested that coping strategies, social skill therapy, and programs are essential considerations for the teenage population's promising futures [19]. It is high time for healthcare professionals to recognize the illness and raise awareness about ADHD among teachers, parents, and primary care physicians to prevent the disorder's social and academic consequences [41].

The research highlights the importance of addressing psychological challenges faced by adolescents, especially during stressful periods such as the current COVID-19 pandemic. Coping strategies and social skills therapy have been identified as crucial interventions for promoting a positive future for teenagers. Overall, this discussion highlights the necessity for comprehensive strategies and increased awareness to support the mental well-being of children and adolescents in various environments.

Studies Related to Sexual and Emotional Abuse/Violence and Mental Health

Violence against children and adolescents is a global public health issue that requires special attention globally. In India, psychological, physical, and sexual violence was experienced by 52.4%, 25.1%, and 12.7% of adolescents, respectively [43]. Besides, very few studies focus on sexual and emotional abuse in developing countries like India. The lifetime prevalence of emotional (85.7%) and sexual (23.8%) abuse was high among adolescents. In terms of age prevalence, psychological violence was more common among older adolescents (aged 17-18 years) than among younger adolescents (15-16 years) [43].

Furthermore, in terms of gender, a greater proportion of males than females reported being victims of abuse [39]. Some studies also showed that male gender, low socioeconomic level, regular use of substance abuse by family members at home, and having other challenges at school all considerably increase the likelihood of abuse [39]. Since the children were less likely to disclose abuse if they liked attending school and always felt safe there. The findings show the urgent need to deal with and minimize abuse in the educational setting [39]. The current study highlights the utmost necessity for providing personal counseling services to teenagers who have experienced abuse. These services aid in the recovery of their psychological wounds, allowing them to positively move forward in their lives with renewed optimism and ambitions. The discussion further stresses the essentiality of implementing comprehensive measures to combat violence against children and adolescents, including preventive measures, support resources in educational institutions, and specialized counseling services for those impacted.

The review suggested a high prevalence of mental health issues among school children and adolescents in India, which is alarming for the future. India has limited mental health services for children and adolescents. Most of these services are limited to urban settings. A significant gap exists in the prevention of mental illness, promotion, and early intervention of mental health activities. Access to mental health services is inadequate; they are not offered early enough and are only available to a small proportion of children and adolescents. The mental health intervention should not solely focus on training teachers but also on involving other mental health professionals who may work with school children and adolescents. There is a requirement for more community-based interventions, particularly for school children and adolescents residing in the community. Effective mental health and community agency partnerships and collaboration agreed upon at the senior officer level by local, state, and national levels and educational institutions are necessary to deliver mental health services. The school Mental Health initiatives and child policy for mental health have provided tremendous opportunities to improve the mental health status of children and adolescents. The effectiveness of child mental health intervention will undoubtedly help in the treatment and rehabilitation of children and adolescents with mental health issues.

## Conclusions

The current systematic review of 31 studies has focused on mental health issues among school children and adolescents in India. Our review highlighted the high prevalence of depression, behavioral and emotional problems, anxiety, psychological distress, internet addiction, and other mental health issues in school children and adolescents. Moreover, the findings of the review have been synthesized from different parts of the country and at various age groups. Hence, there is an urgent need to strengthen and reinforce school-based mental health screening programs with an integrated approach to recognize mental health symptoms and raise awareness about mental health issues among teachers, parents, and stakeholders in school and community settings. The review also suggested that healthcare professionals collaborate with parents and teachers for training to provide prompt intervention and family-focused strategies to deal with problems more effectively. Furthermore, it also indicates the need for advocacy for the welfare of school children and adolescents and the provision of skilled counselors who can collaborate with mental health professionals.

The inclusion of proficient counselors working alongside mental health experts is emphasized as an essential element in providing comprehensive support for mental health. This conversation underscores the urgency for a unified and multifaceted strategy to tackle the varied mental health issues faced by students and young adults in India.

## Appendices

**Table 3:** PRISMA 2020 for abstracts checklist From: Page MJ, McKenzie JE, Bossuyt PM, Boutron I, Hoffmann TC, Mulrow CD, et al. The PRISMA 2020 statement: an updated guideline for reporting systematic reviews. BMJ 2021;372:n71. doi: 10.1136/bmj.n71 PRISMA: Preferred Reporting Items for Systematic Reviews and Meta-Analyses

Section and Topic	Item #	Checklist item	Reported (Yes/No)
TITLE			
Title	1	Identify the report as a systematic review.	Yes
BACKGROUND			
Objectives	2	Provide an explicit statement of the main objective(s) or question(s) the review addresses.	Yes
METHODS			
Eligibility criteria	3	Specify the inclusion and exclusion criteria for the review.	Yes
Information sources	4	Specify the information sources (e.g. databases, registers) used to identify studies and the date when each was last searched.	Yes
Risk of bias	5	Specify the methods used to assess risk of bias in the included studies.	No
Synthesis of results	6	Specify the methods used to present and synthesise results.	No
RESULTS			
Included studies	7	Give the total number of included studies and participants and summarise relevant characteristics of studies.	Yes
Synthesis of results	8	Present results for main outcomes, preferably indicating the number of included studies and participants for each. If meta-analysis was done, report the summary estimate and confidence/credible interval. If comparing groups, indicate the direction of the effect (i.e. which group is favoured).	Yes
DISCUSSION			
Limitations of evidence	9	Provide a brief summary of the limitations of the evidence included in the review (e.g. study risk of bias, inconsistency and imprecision).	No
Interpretation	10	Provide a general interpretation of the results and important implications.	Yes
OTHER			
Funding	11	Specify the primary source of funding for the review.	NA
Registration	12	Provide the register name and registration number.	NA

**Table 4:** PRISMA 2020 checklist From: Page MJ, McKenzie JE, Bossuyt PM, Boutron I, Hoffmann TC, Mulrow CD, et al. The PRISMA 2020 statement: an updated guideline for reporting systematic reviews. BMJ 2021;372:n71. doi: 10.1136/bmj.n71 PRISMA: Preferred Reporting Items for Systematic Reviews and Meta-Analyses

Section and Topic	Item #	Checklist item	Reported on page #
TITLE			
Title	1	Identify the report as a systematic review.	1
ABSTRACT			
Abstract	2	See the PRISMA 2020 for Abstracts checklist.	1
INTRODUCTION			
Rationale	3	Describe the rationale for the review in the context of existing knowledge.	3
Objectives	4	Provide an explicit statement of the objective(s) or question(s) the review addresses.	3
METHODS			
Eligibility criteria	5	Specify the inclusion and exclusion criteria for the review and how studies were grouped for the syntheses.	Page 4
Information sources	6	Specify all databases, registers, websites, organisations, reference lists and other sources searched or consulted to identify studies. Specify the date when each source was last searched or consulted.	3-4
Search strategy	7	Present the full search strategies for all databases, registers and websites, including any filters and limits used.	3
Selection process	8	Specify the methods used to decide whether a study met the inclusion criteria of the review, including how many reviewers screened each record and each report retrieved, whether they worked independently, and if applicable, details of automation tools used in the process.	4
Data collection process	9	Specify the methods used to collect data from reports, including how many reviewers collected data from each report, whether they worked independently, any processes for obtaining or confirming data from study investigators, and if applicable, details of automation tools used in the process.	4
Data items	10a	List and define all outcomes for which data were sought. Specify whether all results that were compatible with each outcome domain in each study were sought (e.g. for all measures, time points, analyses), and if not, the methods used to decide which results to collect.	Table 1
	10b	List and define all other variables for which data were sought (e.g. participant and intervention characteristics, funding sources). Describe any assumptions made about any missing or unclear information.	NA
Study risk of bias assessment	11	Specify the methods used to assess risk of bias in the included studies, including details of the tool(s) used, how many reviewers assessed each study and whether they worked independently, and if applicable, details of automation tools used in the process.	5
Effect measures	12	Specify for each outcome the effect measure(s) (e.g. risk ratio, mean difference) used in the synthesis or presentation of results.	NA
Synthesis methods	13a	Describe the processes used to decide which studies were eligible for each synthesis (e.g. tabulating the study intervention characteristics and comparing against the planned groups for each synthesis (item #5)).	6
	13b	Describe any methods required to prepare the data for presentation or synthesis, such as handling of missing summary statistics, or data conversions.	6
	13c	Describe any methods used to tabulate or visually display results of individual studies and syntheses.	6
	13d	Describe any methods used to synthesize results and provide a rationale for the choice(s). If meta-analysis was performed, describe the model(s), method(s) to identify the presence and extent of statistical heterogeneity, and software package(s) used.	6
	13e	Describe any methods used to explore possible causes of heterogeneity among study results (e.g. subgroup analysis, meta-regression).	NA
	13f	Describe any sensitivity analyses conducted to assess robustness of the synthesized results.	NA
Reporting bias assessment	14	Describe any methods used to assess risk of bias due to missing results in a synthesis (arising from reporting biases).	Table 2
Certainty assessment	15	Describe any methods used to assess certainty (or confidence) in the body of evidence for an outcome.	NA
RESULTS			
Study selection	16a	Describe the results of the search and selection process, from the number of records identified in the search to the number of studies included in the review, ideally using a flow diagram.	Figure 1
	16b	Cite studies that might appear to meet the inclusion criteria, but which were excluded, and explain why they were excluded.	NA
Study characteristics	17	Cite each included study and present its characteristics.	Table 1
Risk of bias in studies	18	Present assessments of risk of bias for each included study.	Table 2
Results of individual studies	19	For all outcomes, present, for each study: (a) summary statistics for each group (where appropriate) and (b) an effect estimate and its precision (e.g. confidence/credible interval), ideally using structured tables or plots.	NA
Results of syntheses	20a	For each synthesis, briefly summarise the characteristics and risk of bias among contributing studies.	NA
	20b	Present results of all statistical syntheses conducted. If meta-analysis was done, present for each the summary estimate and its precision (e.g. confidence/credible interval) and measures of statistical heterogeneity. If comparing groups, describe the direction of the effect.	NA
	20c	Present results of all investigations of possible causes of heterogeneity among study results.	NA
	20d	Present results of all sensitivity analyses conducted to assess the robustness of the synthesized results.	NA
Reporting biases	21	Present assessments of risk of bias due to missing results (arising from reporting biases) for each synthesis assessed.	NA
Certainty of evidence	22	Present assessments of certainty (or confidence) in the body of evidence for each outcome assessed.	NA
DISCUSSION			
Discussion	23a	Provide a general interpretation of the results in the context of other evidence.	16
	23b	Discuss any limitations of the evidence included in the review.	NA
	23c	Discuss any limitations of the review processes used.	NA
	23d	Discuss implications of the results for practice, policy, and future research.	24
OTHER INFORMATION			
Registration and protocol	24a	Provide registration information for the review, including register name and registration number, or state that the review was not registered.	NA
	24b	Indicate where the review protocol can be accessed, or state that a protocol was not prepared.	NA
	24c	Describe and explain any amendments to information provided at registration or in the protocol.	NA
Support	25	Describe sources of financial or non-financial support for the review, and the role of the funders or sponsors in the review.	NA
Competing interests	26	Declare any competing interests of review authors.	NA
Availability of data, code and other materials	27	Report which of the following are publicly available and where they can be found: template data collection forms; data extracted from included studies; data used for all analyses; analytic code; any other materials used in the review.	NA
